# Comparisons of Measured and Self-Reported Anthropometric Variables and Blood Pressure in a Sample of Hong Kong Female Nurses

**DOI:** 10.1371/journal.pone.0107233

**Published:** 2014-09-15

**Authors:** Yao Jie Xie, Suzanne C. Ho, Zhao Min Liu, Stanley Sai-Chuen Hui

**Affiliations:** 1 Department of Sports Science and Physical Education, the Chinese University of Hong Kong, Hong Kong SAR, China; 2 Division of Epidemiology, The Jockey Club School of Public Health and Primary Care, the Chinese University of Hong Kong, Hong Kong SAR, China; 3 Department of Medicine & Therapeutics, the Chinese University of Hong Kong, Hong Kong SAR, China; The University of Hong Kong, Hong Kong

## Abstract

**Objectives:**

To assess the validity of self-reported weight, height, body mass index (BMI), waist circumference and blood pressure compared with standardized clinical measurements and to determine the classification accuracy in overweight/obesity and central adiposity.

**Methods:**

This pilot study was integrated into a life-course study entitled “Hong Kong Women's Health Study” among 1,253 female nurses in Hong Kong who were aged 35 years to 65 years. Data were collected from self-administered questionnaires that were mailed to the respondents. Of these participants, we obtained the standard body measurements of 144 (11.5%) at our research center. We then compared the self-reported anthropometric variables and blood pressure with the measured data to assess validity based on the level of misreporting, percentage of agreement, consistency, sensitivity and specificity.

**Results:**

The self-reported and measured values were highly correlated in terms of anthropometry and blood pressure (correlation coefficients ranged from 0.72 to 0.96). Height was overestimated at an average of 0.42 cm, and waist circumference was underestimated at 2.33 cm (both P<0.05), while no significant differences were observed from weight, blood pressure and BMI (all P>0.05). The proportions of overweight, obesity, and central adiposity by self-reported data did not vary greatly from the measured data (all P>0.05). The self-reporting resulted in correct classifications of BMI, waist circumference, and systolic blood pressure in 85%, 78%, and 87% of women, with corresponding Kappa index values of 0.79, 0.55, and 0.82, respectively. Sensitivity and specificity were 84.6% and 95.7%, respectively, with respect to overweight/obesity detection, whereas those for central adiposity detection were 70.6% and 83.8%, respectively.

**Conclusion:**

In a sample of female Hong Kong nurses, the self-reported measures of height, weight, BMI, waist circumference and blood pressure were generally valid. Furthermore, the classification accuracies of overweight/obesity and central adiposity were acceptable.

## Introduction

Height, weight, and waist circumference are common anthropometric variables used in Epidemiology studies. They represent the physical characteristics of a person and reflect the biological, genetic, and environmental characteristics of a population. The body mass index (BMI, kg/m^2^), which is calculated from weight (kilogram) divided by the square of the height (meter), is an indicator of population health because of its measurement function for general obesity [1], [2]. Similarly, waist circumference is an important indicator for measuring central adiposity [3]. In addition, as one of the four vital signs of the human body, blood pressure is an important clinical indicator of hypertension, which in turn predicts the risk of cardiovascular diseases [4]. Many population-based epidemiological studies obtained these anthropometric variables and blood pressure data through self-reporting because of their low cost and high efficiency and convenience. However, a number of studies [1], [5]–[7] indicate that people tend to overestimate height and underestimate weight, thus resulting in the underestimation of BMI [7], [8]. As a result, the prevalence of overweight and obesity is underestimated and the relationships between obesity and health outcomes may be biased [9]. The self-report biases of anthropometric variables and blood pressure are typical in literature [1], [10]–[14]. Furthermore, previous studies suggest that women are more likely than men to underestimate weight [5], [15]. Based on a systematic review, however, women with BMIs less than 20.0 kg/m^2^ overestimated weight [1]. This paradoxical self-reporting behavior in women may be attributed to social, cultural, and psychological factors. Thus, the accuracy of the self-reported measures needs to be determined further [16].

To our knowledge, comparable studies have not been conducted on the Chinese adult population in Hong Kong to validate self-reported anthropometric measures. We thereby conducted the present study with the aims to examine the validity of self-reported weight, height, BMI, waist circumference, and blood pressure by comparison with face-to-face measured values in a sample of adult women in Hong Kong and to determine the classification accuracy in overweight/obesity and central adiposity according to self-reported values.

## Methods

### Study design and sample

In 2010, we launched a life-course epidemiology study called, “Hong Kong Women's Health Study”. This study investigated the relationships between early life exposures (e.g., birth weight, physical activity in adolescence) and later life health outcomes (e.g., adult obesity, hypertension). The present validation study was integrated into the Hong Kong Women's Health Study as a pilot study. The study population was composed of female Chinese nurses in Hong Kong who were aged 35 years to 65 years. The Association of Hong Kong Nursing Staff helped with the systematic sampling of total 8320 eligible nurses from their membership database. Packages were mailed to the home addresses of the selected nurses, and these packages contained a questionnaire, an informed consent form, an introduction leaflet with a reply slip, a paper tape measure, and a pre-paid return envelope [17]. The questionnaire was divided into six sections: personal information, work status, lifestyle information (from adolescence to the present), reproductive information, health status and diseases encountered, and dietary habits. It contained a total of 33 questions composed of 153 items and was printed on a 4-double page booklet with a cover page that provided a brief introduction of the study.

Finally, 1,253 nurses who returned valid questionnaires were recruited. To validate the self-reported anthropometrics and blood pressure in the questionnaire, we asked the potential responders whether they wanted to participate in the scheduled face-to-face body measurement described in the mailed survey. Those who were willing could tick the corresponding box in the reply slip and return it together with the questionnaire. Once we received the valid reply slips, we contacted the participants through telephone or/and email immediately and invited them to visit our research center for taking body measurements within one month. This minimized time duration prevented anthropometric variations as a result of a long delay in between the gathering of self-reported values and the measurements. The study protocol was approved by the Research Ethics Committee of the Chinese University of Hong Kong. A signed statement of informed consent was obtained from each participant.

### Measurements

Regarding to the self-reported anthropometrics and blood pressure, participants were asked to measure and record the value according to the written instructions. The height and waist circumference could be reported in either centimeter or inch, and the weight could be reported in either pounds or kilograms. All data were converted to metric units (meter or kilogram) for analysis. The guideline for measuring waist circumference was printed on the introduction leaflet. Participants were required to use the complimentary paper tape measure to measure their waist circumference. For the blood pressure, a note was printed in the questionnaire that reminded them to provide the value from the most recent body check. If a sphygmomanometer was available, they were asked to measure their blood pressure at least twice, with the help of other professional person, and write down the mean blood pressure on the questionnaire.

The face-to-face measurement procedures for all anthropometrics were based on standard protocols. All measurements were conducted by the investigator and a trained research assistant. Height was measured to the nearest 0.1 cm by a stadiometer. First, we confirmed that the surface on which the scale was placed was horizontal and made adjustments to ensure that the upper part of the measuring rod was straight and vertical. Prior to measurement, participants were required to remove hair ornaments, shoes, and heavy outer garments. They were then advised to stand straight and look straight ahead. Their backs were pressed to the height rule, with their shoulders and arms drooping naturally. Furthermore, they were requested to keep their feet together. The research assistant ensured that the backs of the heads, backs, buttocks, calves, and heels of the participants were touching the rule. The slide of the measuring rod was gradually lowered until the hairs of the participants were flattened and the rule could not move any further. The investigator then wrote down the readings.

A standardized weighing scale was used to measure body weight to the nearest 0.1 kg. This scale was also placed on a horizontal hard-floor surface. Prior to measurements, participants removed heavy outer garments such as jackets, coats, skirts, shoes, socks, hats, watches, necklaces, and other carry-on items. They then stood at the center of the machine platform to prevent the unbalanced distribution of weight between the two feet. Weight was then recorded when the arrow no longer wiggled and was aligned steadily. BMI was then calculated as weight in kilograms divided by the square of height in meters (BMI  =  kg/m^2^).

Waist circumference was measured by a standardized tape measure. Prior to measurement, participants were requested to loosen their belts and empty their pockets. They then stood with their feet apart at approximately 12 cm–15 cm to ensure that weight was equally distributed along each leg. The measuring tape was held firmly by the observer to ensure its horizontal position as it was wound around the body. However, it should be held loosely enough to accommodate one finger between the tape and the participant's body. The measuring position was at a level midway between the lower rib margin and the iliac crest. The measurement was recorded after the participant exhaled gently in normal breathing to avoid the contraction of abdominal muscles. Measurements were recorded to the nearest 0.1 cm.

Every measurement mentioned above was conducted twice, and the average value was calculated. If the two measurements differed by >2 cm or >2 kg, a third measurement was conducted and the mean of the final two measurements was calculated.

Blood pressure was measured to the nearest 0.1 millimeter of mercury (mmHg) by the observer using a mercury sphygmomanometer and an appropriately sized cuff. Participants who were currently taking anti-hypertension medicine were asked to not take it before coming to the center for body measurements. Measurements were taken while the participants were seated and given at least five minutes of rest. The right arms of the participants were positioned on a table, with their palms up at chest height. Every participant was measured twice, and the mean of two measurements was computed. If the two measurements varied by >20 mmHg, a third measurement was conducted and the mean of two similar measured values was calculated. If participants were found to have elevated blood pressure levels (diastolic ≥90 mmHg or systolic ≥140 mmHg), they were asked to remain at rest for at least another five minutes before the second and third readings were taken. The mean of the two lower measurements was then computed. Hypertension was defined as either a systolic blood pressure of ≥140 mmHg or a diastolic blood pressure of ≥90 mmHg [18].

### Statistical analysis

Data were presented as mean (standard deviation) or proportion as appropriate. To verify the representativeness of the participants in validity examinations, we examined the differences in demographic characteristics and key variables between subjects who participated in the validity examination and the total sample by a one-sample *t*-test for continuous variables and chi-square tests for nominal and ordinal variables. The mean differences between the measured variables and self-reported variables were investigated by paired-samples *t*-tests. The degree of correlation was measured by Pearson correlation analysis. BMI and waist circumference were used to identify obese subjects among participants. The BMI results were divided into four categories: underweight (<18.5 kg/m^2^), normal weight (18.5 kg/m^2^–<23.0 kg/m^2^), overweight (23.0 kg/m^2^–<25.0 kg/m^2^), and obese (≥25.0 kg/m^2^) [19]. Central adiposity was defined as waist circumference ≥80 cm according to the standard of the World Health Organization for Asian populations [19]. McNemar tests were conducted to determine the proportion differences between the measured and self-reported binary variables, namely, 1) overweight, 2) obese, and 3) central adiposity. For the four categories of BMI and two categories of waist circumference, the percentage of agreements between self-reported and measured data were calculated and the Kappa test was used to assess the degree of consistency. Blood pressure was also divided into several categorical groups to determine the agreement and consistency. Sensitivity [true positives/(true positives + false negatives)] and specificity [true negatives/(true negatives + false positives)] of overweight/obese and central adiposity were calculated. The true measure was represented by the data measured face to face. Moreover, Bland-Altman plot [20] was used to describe the detail of agreement, providing mean agreement and 95% limits of agreement. All analyses were performed using SPSS 19.0 software, and a P-value of <0.05 was regarded as statistically significant.

## Results

A total of 216 nurses were willing to participate in the body check, and 144 nurses (11.5% of the total sample; 144/1253) successfully completed the face-to-face body measurements (Supplementary S1). The demographical information, anthropometric indicators, and lifestyle factors of the validation and the total samples are shown in Table 1. The validation and the total samples did not differ significantly in height, BMI (calculated by self-reported height and weight), blood pressure, drinking habits, physical activity, marital status, and monthly family income (all P values >0.05); while participants in validation sample were significantly older, heavier, more likely to have central adiposity, and reported relatively lower education levels (all P values <0.05).

**Table 1:** Characteristics of the validation study sample (*n* = 144) and the total sample (*n* = 1253), 2011, Hong Kong.

Participant characteristics	Validation sample (*n* = 144)	Total sample (*n* = 1253)	P value
Age (year), mean (SD)	47.9 (7.9)	45.6 (7.6)	0.000
Height (cm), mean (SD)	158.9 (5.7)	158.4 (5.5)	0.236
Weight (kg), mean (SD)	56.5 (8.1)	55.1 (8.1)	0.034
BMI (kg/m2), mean (SD)	22.3 (3.0)	22.0 (3.0)	0.171
BMI categories, n (%)			0.701
Underweight (BMI <18.5)	8 (5.6)	94 (7.6)	
Normal (BMI 18.5–<23.0)	87 (60.4)	765 (61.8)	
Overweight (BMI 23.0–<25.0)	29 (20.1)	213 (17.2)	
Obese (BMI = or >25.0)	20 (13.9)	166 (13.4)	
Waist circumference (cm), mean (SD)	77.6 (7.5)	75.3 (8.4)	0.004
Waist circumference categories, n (%)			0.001
Central adiposity - Yes (> = 80 cm)	60 (42.3)	349 (28.4)	
Central adiposity - No (<80 cm)	82 (57.7)	881 (71.6)	
Systolic blood pressure, mean (SD)	111.3 (12.6)	113.5 (13.4)	0.778
Diastolic blood pressure, mean (SD)	71.7 (9.7)	70.4 (9.9)	0.451
Current drinker, n (%)			0.594
Yes	132 (92.3)	1158 (93.5)	
No	11 (7.7)	81 (6.5)	
Physical activity, n (%)			0.280
Average or lower	79 (55.2)	746 (60.1)	
Higher than average	64 (44.8)	495 (39.9)	
Marital status, n (%)			0.831
Married	104 (72.2)	916 (73.3)	
Divorced/separated/widowed	12 (8.3)	87 (7.1)	
Single	28 (19.5)	247 (19.8)	
Education level, n (%)			0.006
Primary and secondary school	50 (34.7)	347 (27.9)	
Advanced level curriculum	18 (12.5)	86 (6.9)	
Post-secondary education	60 (41.7)	578 (46.4)	
Masters and above	16 (11.1)	234 (18.8)	
Family income (HK$/month), n (%)			0.080
<20000	8 (6.0)	35 (3.0)	
20000–40000	51 (38.3)	372 (31.8)	
40001–60000	41 (30.8)	415 (35.3)	
>60000–100000	33 (24.9)	352 (29.9)	

Table 2 indicates the mean values of the measured and self-reported anthropometric variables and blood pressure in the validation sample, as well as the mean differences in paired variables and their correlation coefficients. All paired variables were significantly and highly correlated (all P values <0.001). The correlation coefficients ranged from 0.72 to 0.96, most of them were higher than 0.90, except waist circumference (correlation coefficient: 0.78) and diastolic blood pressure (correlation coefficient: 0.72). On average, the self-reported values did not vary significantly from the measured values in terms of weight, BMI, and blood pressure (all P values >0.05). However, self-reported height was higher than the measured data at an average of 0.42 cm, and self-reported waist circumference was approximately 2.33 cm less than the measured value (both P values <0.05).

**Table 2:** Mean values of measured and self-reported variables, mean differences, and the correlations between paired variables, 2011, Hong Kong (*N* = 144).

	Mean (SD)		Mean difference (SD)	95% CI of the difference	Correlation R
	Measured values	Self-reported values			
Height (cm)	158.51 (5.68)	158.93 (5.86)	0.42 (1.58)**	0.16, 0.68	0.96***
Weight (kg)	56.58 (8.22)	56.56 (8.14)	−0.02 (3.31)	−0.59, 0.52	0.92***
BMI (kg/m^2^)	22.52 (3.07)	22.39 (2.95)	−0.14 (1.36)	−0.36, 0.09	0.90***
Waist circumference (cm)	79.90 (8.03)	77.57 (7.59)	−2.33 (5.21)***	−3.19, −1.46	0.78***
Systolic blood pressure (mmHg)	110.91 (13.61)	111.28 (12.62)	0.37 (5.78)	−0.67, 1.40	0.91***
Diastolic blood pressure (mmHg)	72.41 (9.65)	71.74 (9.67)	−0.67 (7.28)	−1.97, 0.63	0.72***

** P<0.01,

*** P<0.001.

Table 3 shows the validity indicators of the classification for BMI, waist circumference, and blood pressure categories according to the measured and self-reported data. When the participants were categorized into underweight, normal weight, overweight, and obese groups based on BMI, the proportions of the overweight and obese groups as estimated from self-reported data were 1.4 and 0.7 percentage points less, respectively, than those estimated from measured data (20.1% versus 21.5% and 13.9% versus 14.6%). The differences were statistically insignificant according to the McNemar test (both P>0.05). When the participants were categorized into central adiposity and normal groups by waist circumference, the central adiposity proportion based on self-reported waist circumference was 5.6 percentage points less than that estimated from measured data (42.3% versus 47.9%). Nonetheless, this difference was also insignificant (McNemar test, P>0.05). The self-reported weights, heights, and waist circumferences resulted in the correct classification of BMI categories and central adiposity in 85% and 78% of the women (Table 3, overall agreement: 84.7% and 77.5%, respectively). When the various classes of self-reported blood pressure were compared to their measured values, the overall agreements of systolic and diastolic blood pressure were 87% and 72%, respectively. Given the categorical variables of BMI, waist circumference and blood pressure, the weighted Kappa coefficients ranged from 0.55 in terms of waist circumference to 0.82 for systolic blood pressure (Table 3). With regard to overweight/obesity detection (Table 3), 8 out of 52 truly overweight/obese participants were misclassified to under/normal weight categories by self-reporting. Furthermore, 5 of 92 truly under/normal weight participants were misclassified. The resultant sensitivity and specificity were 84.6% (44/52) and 95.7% (87/92), respectively. In terms of central adiposity detection, 20 participants were false negative, whereas 12 were false positive, the sensitivity and specificity were 70.6% (48/68) and 83.8% (62/74), respectively.

**Table 3:** Validity indicators for categorical variables BMI, waist circumference, and blood pressure, including the classification percentage and weighted kappa coefficient of the self-reported and clinical body measures, 2011, Hong Kong (*N* = 144).

Categorical variables	Self-reported values		Measured values		Agreement (%)		Kappa ^a^	
	n	%	n	%	%	95% CI	K	95% CI
BMI classification					84.7	77.6, 90.0	0.79	0.71, 0.88
Underweight (<18.5)	8	5.6	8	5.6				
Normal (18.5–23.0)	87	60.4	84	58.3				
Overweight (23.0–25.0)	29	20.1	31	21.5				
Obesity (≥25.0)	20	13.9	21	14.6				
WC classification					77.5	69.5, 83.9	0.55	0.41, 0.68
Normal (<80 cm)	82	57.7	74	52.1				
Central adiposity (≥80 cm)	60	42.3	68	47.9				
SBP classification					86.8	79.9, 91.7	0.82	0.74, 0.89
SBP <100	19	13.2	26	18.1				
SBP: 100–119	88	61.1	78	54.2				
SBP: 120–139	33	22.9	35	24.3				
SBP ≥140	4	2.8	5	3.5				
DBP classification					71.5	63.3, 78.6	0.65	0.54, 0.75
DBP <70	56	38.9	54	37.5				
DBP: 70–79	50	34.7	54	37.5				
DBP: 80–89	24	16.7	27	18.8				
DBP ≥90	14	9.7	9	6.2				

WC: waist circumference; SBP: systolic blood pressure; DBP: Diastolic blood pressure.

a. Kappa coefficients with linear weighting were calculated for these ordinal variables by kappa_LW_  =  (P_observed_ – P_expected_)/(1− P_expected_).

**Figure 1:**
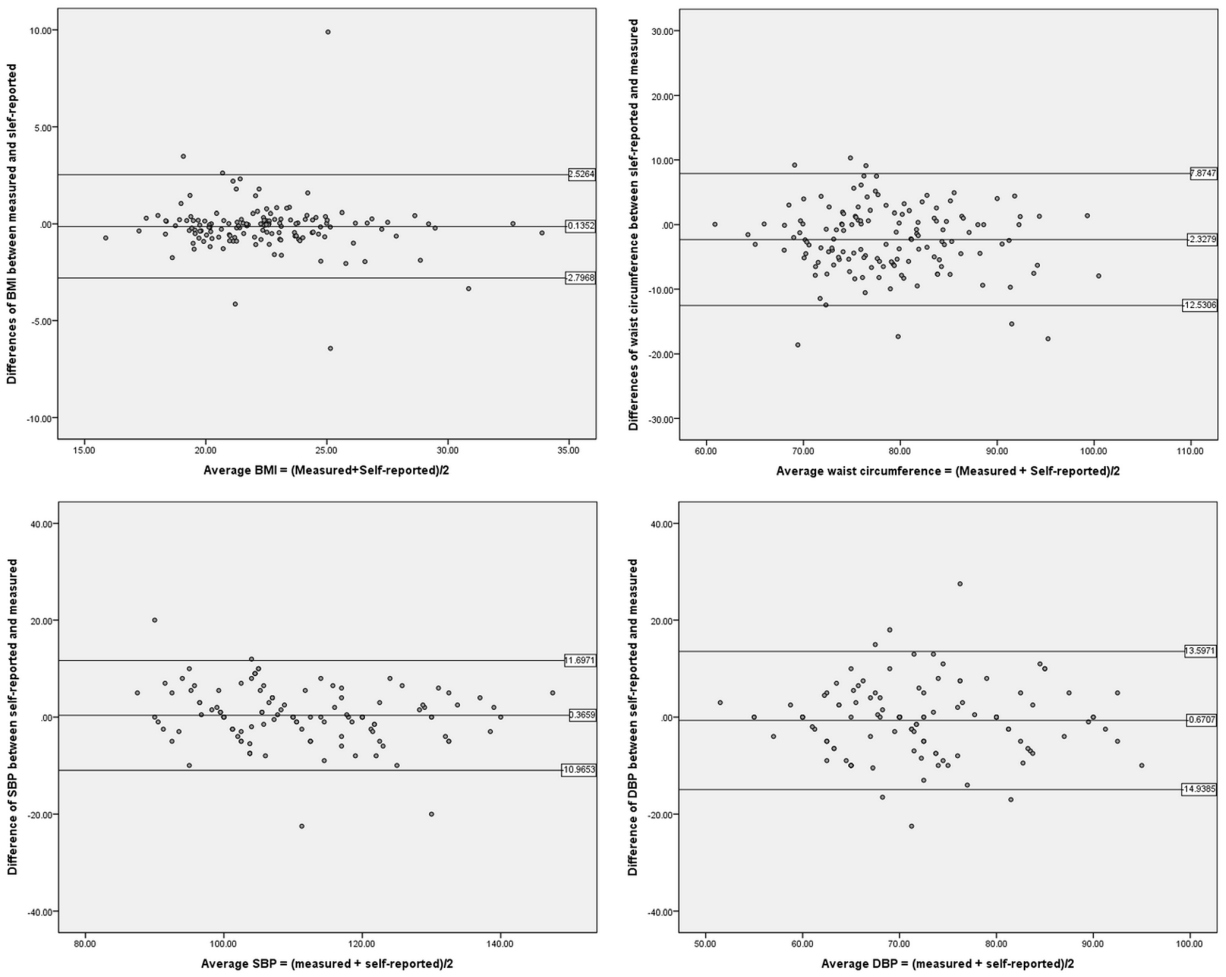
Bland-Altman plot of the differences between the self-reported and measured values of BMI, waist circumference and blood pressure versus the mean of self-reported and measured values. Horizontal lines represent mean difference and 95% limits of agreement.

Figure 1 presents the Bland-Altman plot of the differences between the self-reported and measured values of BMI, waist circumference, and blood pressure versus the average values obtained by (self-reported + measured)/2. The horizontal lines represent the mean difference and 95% limits of agreement. As shown in this figure, variability is relatively random, which indicates that the self-reported and measured values were highly consistent across all variables.

## Discussion

Our study indicated that middle-aged female nurses in Hong Kong could accurately report their weight and blood pressure values but that they underestimated waist circumference and overestimated height. These biases from self-reported values resulted in a slight misclassification of overweight/obesity and central adiposity. Nonetheless, the overall agreements with respect to the reporting of accurate values were reliable and acceptable.

Our findings are consistent with those of previous studies [10], [14], [21]–[25] that self-reported height and weight were highly correlated with measured values. Similar results [12], [26]–[28] were obtained with regard to waist circumference and blood pressure. Despite the high correlations, however, self-reported values retained some bias. Height was overestimated and waist circumference was underestimated, it was termed “flat slope syndrome” that high values tend to be underestimated, low values are overestimated [29]. Individual's self-knowledge on body size is likely to be influenced by local culture, as individuals at either end of the body size continuum potentially tailor their self-reports toward some socially desirable 'ideal' body size [30]. Height was also misreported in previous studies [7], [10], [21], [25], [31], but the extent observed in our study is smaller than that in most of the studies on general adult populations. In these studies, the mean difference in height (calculated as self-reported height minus measured height) ranged from 0.4 cm to 7.5 cm [1], [7], [10], [21], [25]. Moreover, weight was not significantly underestimated in our study (at only −0.02 kg). This underreporting of weight is far less than that in previous studies, which ranged from 0.5 kg to 6.5 kg [1], [7], [10], [21], [25]. Engstrom *et al.* [5] reviewed 34 studies on self-reported height and weight in women and noted that in 21 studies, women overestimated their height. In all studies, women underestimated their weight. However, the women in our study reported their weight more accurately than those in previous studies. Subsequently, the BMI calculation was highly accurate. In our study, the mean BMIs of the measured and self-reported data did not differ significantly. Hence, a correct classification in BMI categories was achieved at 85% and a Kappa coefficient of 0.79. These findings were similar to those of a previous study that reported a precise classification of 83.4% [7]. Thus, our study reflects reliable and acceptable [32] results. With respect to overweight/obesity detection by self-reported BMI in our study, the sensitivity and specificity were 84.6% and 95.7%, respectively, which were similar to those reported by the aforementioned study (sensitivity 81%, specificity 97%) [7]. Our sensitivity value was also far higher than that obtained in a sample from a Western community (73% for women) [33].

In our study, the accuracy of self-reported blood pressure was also acceptable. The mean differences between measured and self-reported values were slight, which is consistent with the Harvard Nurses' Health Study [28]. However, the biggest difference was found in waist circumference. This misreporting was consistent with that by a large cohort study [31], which concluded that waist measurements were misreported to a greater extent than weight and height. In our study, the accuracy of self-reported obesity as defined by BMI was higher than that of self-reported central adiposity. Despite the extent of bias in reported waist circumference, the degree of classification to the correct categories was 78% based on self-reported values in our study, and the Kappa values showed an acceptable consistency.

Some limitations of the present study must be addressed. First, the relatively small sample size for validation may lower the study power and representativeness. In addition, the nurse sample may limit the generalizability of the results. Second, due to the limited manpower and material resources, we did not control for the circadian variation in blood pressure measurement, which could present a possible confounding effect on the measurement bias. But we suggest that it unlikely to substantially affect the overall results, because it was random error when the time frame for self-measurement was no limit. Third, we recruited the study subjects through convenience sampling in the validity examination, only those who indicated their willingness to participate were included. Hence, selection bias may be present to some extent because the behavior decisions of a person may vary with social-demographical status. Our results also indicated that the validation sample was older, heavier, and less educated than the total sample. Despite of these differences, the two samples did not differ significantly in terms of other key variables. Several studies [10], [12], [21], [31], [34] have reported that age affects the accuracy of self-reporting; older people self-report data with lower validity than young people. In our study, the subjects in the entire sample were younger than those in the validation sample, we believe that the self-reported bias is small throughout the entire study sample.

Despite these limitations, no comparable studies have been conducted in Hong Kong adult populations to our knowledge. We found that female nurses in Hong Kong reported their height, weight, and waist circumference more accurately than the participants in some occidental studies. This higher accuracy may be ascribed to the professional medical background of the nurses in our study population and to the personality or cultural characteristics of Hong Kong women. A similar finding was reported in Japan [24], which is another East Asian nation. In conclusion, the self-reported height, weight, BMI, waist circumference, and blood pressure in this population of Hong Kong female nurses can be considered as general valid. Furthermore, the classification accuracies of overweight/obesity and central adiposity were acceptable. However, the slight misreporting of height and waist circumference reminds the importance to carefully interpret data in scientific studies when self-reported indicators are used.

## Supporting Information

Supplementary S1 **Data of the 144 subjects who participated in the face-to-face body measurement.**
(SAV)
